# The Role of Extracellular Vesicles in Systemic Lupus Erythematosus

**DOI:** 10.3389/fcell.2022.835566

**Published:** 2022-03-02

**Authors:** Chenghui Zheng, Lin Xie, Haihong Qin, Xiao Liu, Xi Chen, Fan Lv, Li Wang, Xiaohua Zhu, Jinhua Xu

**Affiliations:** Department of Dermatology, Huashan Hospital, Fudan University, Shanghai, China

**Keywords:** exosome, systemic erythematosus lupus, lupus nephritis, intercellular communication, research progress, extracellular vesicle

## Abstract

Extracellular Vesicles (EVs) are small vesicles that can be actively secreted by most cell types into the extracellular environment. Evidence indicates that EVs can carry microRNAs (miRNAs), long non-coding RNAs (lncRNAs), tRNA-derived small RNAs (tsRNAs), proteins, and lipids to target cells or tissue organizations. Latest studies show that EVs play a vital role in the immune modulation and may contribute to the pathogenesis of autoimmune diseases. Systemic lupus erythematosus (SLE) is a common autoimmune disease characterized by abnormal T cell activation and sustained production of autoantibodies against self-antigens, resulting in inflammation and damage to multiple systems. Pathogenic mechanisms of SLE, however, are still not well understood. In this review, we summarize the latest research advances on the functions and mechanisms of EVs, and its role in the pathogenesis, diagnosis, and treatment of SLE.

## 1 Introduction

Systemic lupus erythematosus (SLE) is a multisystem autoimmune disease characterized by loss of tolerance and sustained production of autoantibodies against self-antigens that form immune complex deposits (Crispin et al., 2010). The prevalence of SLE varies from 30/100,000 to 50/100,000, and the disease is more common in women of childbearing age. SLE is hard to diagnose due to its complex pathogenesis and variable clinical symptoms. Most patients are diagnosed based on the 1997 American College of Rheumatology classification criteria, and the disease activity is assessed based on the SLE Disease Activity Index (SLEDAI). Nevertheless, it is not always effective. At present, a majority of scholars believe that it is the interaction of genetic susceptibility, environment, immunology, and hormone factors that lead to SLE, but the exact mechanism is not clear (Tsokos, 2011). Although non-steroidal anti-inflammatory drugs such as glucocorticoid (GCs), immunosuppressants, and biological agents are commonly used in the treatment of SLE, hurdles such as toxic side effects, the lack of target tissue, and non-response to treatment remain to be crossed (Tsokos, 2011).

First described as “platelet dust” by Peter Wolf in 1967, extracellular vesicles (EVs) are a collective term for phospholipids bilayer structures secreted by cells, which contain microRNAs (miRNAs), long non-coding RNAs (lncRNAs), tRNA-derived small RNAs (tsRNAs), proteins, lipids, and other substances (Raposo and Stoorvogel, 2013). The term “extracellular vesicles (EVs)” includes multiple types of vesicles. Specifically, there exist three main classified subtypes based on their biogenesis, size, and release mechanisms, namely microvesicles (100 nm-1 μm), apoptotic bodies (1–5 μm), and exosomes (30–100 nm in diameter) (Yanez-Mo et al., 2015). Microvesicles (MVs, also called microparticles) are larger than exosomes and pinch directly off from the outer cell membrane (Akers et al., 2013). Microvesicles formation is the result of molecular rearrangements of the plasma membrane regarding phospholipid and cytoskeletal protein composition as well as Ca^2+^ levels (Taylor and Bebawy, 2019). Apoptotic bodies are large structures, which are also produced by direct budding of the membrane and differ from exosomes and MVs as apoptotic bodies are formed only during programmed cell death. They are characterized by the presence of closely packed cellular organelles and fragments of nuleus (Saraste and Pulkki, 2000). Over the past few years, the cutting-edge knowledge about EV research provides insights into new tools for diagnosis, prognosis, and disease activity monitoring, novel therapeutic strategy, and innovative evaluation approaches for treatment effectiveness in SLE (Perez-Hernandez and Cortes, 2015; Perez-Hernandez et al., 2017; Wu et al., 2020; Zhang et al., 2020; Zhao et al., 2020).

**FIGURE 1:**
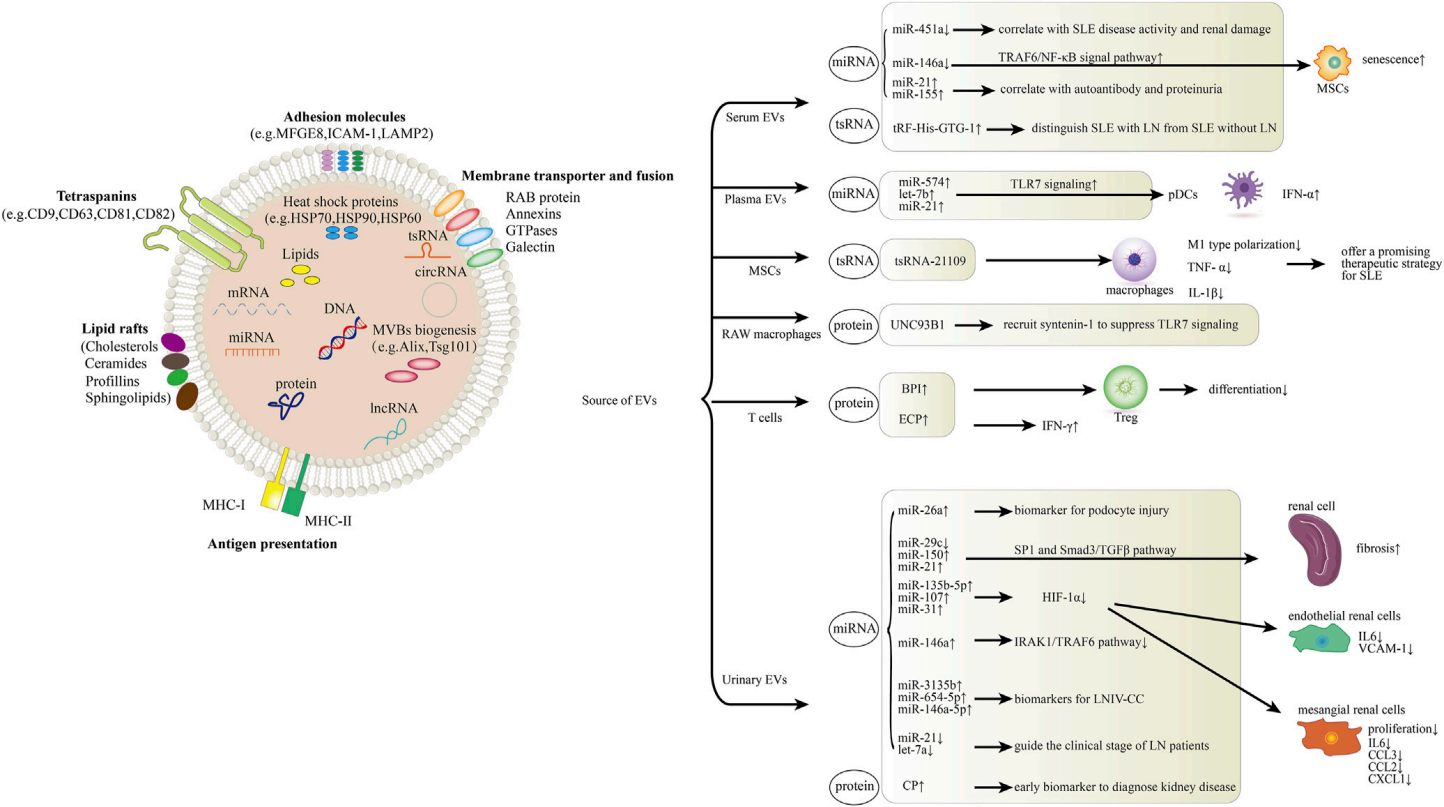
Role of EVs in systemic lupus erythematosus (SLE) and lupus nephritis (LN). The schematic diagram represents how EV components including miRNA, lncRNA, tsRNA and proteins are involved in the pathogenesis of SLE and LN. In serum, EV miR-451a is correlated with SLE disease activity and renal damage. MiR-146a could be internalized into mesenchymal stem cells (MSCs) via circulating EVs and participates in MSCs senescence in SLE patients by targeting TRAF6/NF-*κ*B signal pathway. Serum EV miR-21 and miR-155 expression present correlations with autoantibodies and proteinuria. Levels of serum EV tRF-His-GTG-1 could be used to distinguish SLE with LN from SLE without LN. In plasma, EV miR-574, let-7b and miR-21 activate pDC cells through the TLR7 signaling. MSC-derived EV tsRNA-21109 inhibits the M1-type polarization of macrophages. UNC93B1 can be detected in RAW macrophage-derived EVs, it can recruit syntenin-1 to suppress TLR7 signaling and prevent autoimmunity. Overexpression of BPI in T cell-derived EVs suppresses Treg differentiation and induces EV-mediated inflammation in SLE. ECP overexpression in T cell-derived EVs induces IFN-γ production and tissue inflammation. MiR-26a from urinary EVs can be used as a direct biomarker for podocyte injury. Urinary EV miR-29c, miR-150 and miR-21 promote renal fibrosis through SP1 and Smad3/TGFβ signaling pathway. Urinary EV miR-135b-5p, miR-107 and miR-31 could meliorate renal disease by inhibiting HIF-1α. MiR-146a from urinary EVs negatively regulates inflammation by suppressing the TRAF6 axis. MiR-3135b, miR-654-5p and miR-146a-5p in urinary EVs are candidate biomarkers for Type IV lupus nephritis with cellular crescent (LNIV-CC). Urinary EV let-7a and miR-21 may guide the clinical staging of LN patients. CP, a protein from urinary EVs, could be an early biomarker to diagnose kidney disease.

As the smallest vesicles and probably the most prominently described class of EV, exosomes are ranging from 30–100 nm in diameter, and are released by almost all cell types, including stem cells, T and B lymphocytes, dendritic cells (DCs), macrophages, endothelial cells, neurons, adipocytes, and epithelial cells (Obregon et al., 2009; Mashouri et al., 2019; Rayamajhi et al., 2019). They can be found in a wide range of bodily fluids, such as blood, urine, saliva, breast milk, and in the supernatants of cultured cells after being released into the extracellular environment (Record et al., 2011; Matsumura et al., 2015). Exosome has a lipid bilayer membrane structure and contains bio-reactive macromolecules such as cell-specific proteins, lipids, and nucleic acids, which can protect the coating substances, targeting specific tissues and cells to perform their biological functions. Recently, evidence indicates that exosomes play important roles not only in physiological events, such as intracellular communication, immune modulation, and inflammation, but also in pathological conditions, including autoimmune and cardio-metabolic diseases, as well as development and metastasis of tumors (Shah et al., 2018; Stahl and Raposo, 2019). In this review, we summarize the recent progress of the potential role of exosomes in the pathogenesis, diagnosis, and treatment of SLE (Figure 1 and Table 1). However, there is always a heterogenous population of EVs regardless of the isolation method used. Additionally, none of the involved studies published to date can prove that the isolated fractions are exosomes only. In this context, we utilized the generic term “extracellular vesicles (EVs)” instead of “exosomes” throughout the rest of this survey. It is also in line with the recommended terminology from the international society for extracellular vesicles (ISEV) (Thery et al., 2018).

**TABLE 1:** Diagnostic role of EVs in systemic lupus erythematosus (SLE) and lupus nephritis (LN).

Source of EVs	Isolation method	Candidate markers	Function
Serum EVs	—	MiRNA	—
	ExoQuick Kit	miR-451a↓ Tan et al. (2021)	Correlate with SLE disease activity and renal damage, involved in intercellular communication.
	ExoQuick Kit	miR-146a↓ Dong et al. (2019), Li et al. (2020)	miR-146a is negatively correlated with anti-dsDNA antibodies and participates in mesenchymal stem cells (MSCs) senescence in SLE patients by targeting TRAF6/NF-*κ*B signal pathway
	ExoQuick Kit	miR-21↑ Li et al. (2020)	miR-21 is negatively correlated with anti-SSA/Ro antibodies. miR-21 and miR-155 show positive correlations with proteinuria
	ExoQuick Kit	miR-155↑ Li et al. (2020)	
	—	TsRNA	—
	N.A.	tRF-His-GTG-1↑ Yang et al. (2021)	Can be used to distinguish SLE with LN from SLE without LN.
Plasma EVs	—	MiRNA	—
	Ultracentrifugation	miR-574↑ Salvi et al. (2018)	Activate pDC cells through the TLR7 signaling, allowing them to produce IFN-α and proinflammatory cytokines
		let-7b↑ Salvi et al. (2018)	
		miR-21↑ Salvi et al. (2018)	
Mesenchymal stem cells (MSCs)	—	tsRNA	—
	Cell culture media exosome purification kit	tsRNA-21109 Dou et al. (2021)	Inhibit the M1-type polarization of macrophages
RAW macrophages	Ultracentrifugation	Protein	Recruit syntenin-1 to suppress TLR7 signaling and prevent autoimmunity
		UNC93B1 Majer et al. (2019)	
T cells	ExoQuick Kit	Protein BPI↑ Chuang et al. (2021)	Suppress Treg differentiation and induce EV-mediated inflammation in SLE.
	ExoQuick Kit	ECP↑ Chuang et al. (2022)	Induce IFN-γ production and tissue inflammation
Urinary EVs	Ultracentrifugation	MiRNA miR-26a↑ Ichii et al. (2014)	Can be used as a direct biomarker for podocyte injury
	Ultracentrifugation, miRCURY Exosome Isolation Kit	miR-29c↓ Solé et al. (2015), Solé et al. (2019)	Correlate with renal chronicity (CI) and promote renal fibrosis in LN through SP1 and Smad3/TGFβ signaling pathway
		miR-150↑ Solé et al. (2019)	
		miR-21↑ Solé et al. (2019)	
	miRCURY Exosome Isolation Kit	miR-135b-5p↑ Garcia-Vives et al. (2020)	Meliorate renal disease by inhibiting HIF-1α, can be early markers for predicting LN clinical response
		miR-107↑ Garcia-Vives et al. (2020)	
		miR-31↑ Garcia-Vives et al. (2020)	
	Ultracentrifugation	miR-146a↑ Perez-Hernandez et al. (2015), Perez-Hernandez et al. (2021)	Correlate with lupus activity, proteinuria, and histological features. Negatively regulate inflammation by suppressing the TRAF6 axis
	Ultracentrifugation	miR-3135b↑ Li et al. (2018)	Candidate biomarkers for Type IV lupus nephritis with cellular crescent (LNIV-CC)
		miR-654-5p↑ Li et al. (2018)	
		miR-146a-5p↑ Li et al. (2018)	
	Ultracentrifugation	miR-21↓ Tangtanatakul et al. (2019)	Guide the clinical stage of LN patients
		let-7a↓ Tangtanatakul et al. (2019)	
Urinary EVs	Ultracentrifugation	Protein	Early biomarker to diagnose kidney disease
		CP↑ Gudehithlu et al. (2019)	

↑: increased expression or production; ↓: decreased expression or production.

## 2 Biological Characteristics of EVs

### 2.1 The Biogenesis of EVs

The formation of EVs involves a variety of proteins and transport complexes, and the fusion of primary endocytic vesicles should be the first step of the early endosomes (EEs) formation. Then, two pathways are shown by the EEs. One is that EEs become recycling endosomes, returning to plasma membrane, and the other way is converting into “late endosomes” (LEs)/multivesicular bodies (MVBs) via inward budding of the membrane under the endosomal sorting complex required for transport (ESCRT)-dependent or ESCRT-independent mechanism. Afterwards, LEs are fused with cell membranes, released into the extracellular space under the control of Ras-related proteins in barin (Rab) GTPases and soluble N-ethylmaleimide-sensitive factor attachment protein receptors (SNAREs), which are called EVs (Thery et al., 2002b; Simons and Raposo, 2009; Hessvik and Llorente, 2018).

### 2.2 Isolation and Extraction of EVs

EVs play an essential role in mediating cell communications and participate in the pathological process of multiple diseases. How to extract EVs efficiently with high purity, high recovery, and low cost has become the key to further downstream cell function research. EV samples contain a large number of vesicles or proteins that have similar volume, density or surface charge to EVs, which can interfere with the result of the experiment. A variety of methods have been developed in this regard (Ramirez et al., 2018). Among them, ultracentrifugation is the most widely used protocol and has also evolved as the gold standard for EV separation. There are, however, still some inevitable downsides such as high instrument cost and long extraction time. Moreover, factors (e.g., multiple cleaning, high sample viscosity, etc.) are likely to shape the downstream analyses negatively in an uncertain manner (Momen-Heravi et al., 2012a; Momen-Heravi et al., 2012b). Size exclusion chromatography is a scheme using EV purification columns to separate EVs. It does not require the use of expensive centrifuges, and the obtained EVs have high purity. However, compared to ultracentrifugation, it is more challenging to handle large samples due to the limitations of the purification columns (Koh et al., 2018; Monguio-Tortajada et al., 2019). Faced with bodily fluids and other large-volume samples, ultrafiltration can be perceived as a solution. The principle of ultrafiltration is the same as membrane separation, and it takes less time, but impurities such as other vesicles and proteins tend to block the pores and reduce the extraction efficiency (Cheruvanky et al., 2007; Konoshenko et al., 2018). The above-mentioned traditional extraction methods have multiple drawbacks including low purity, low recovery rate, and low efficiency. Nowadays, a mounting number of new extraction methods are discovered. Lewis et al. (2018) utilized static electricity to adsorb EVs around the positive electrode, which largely improves the purity and specificity of the EVs (Lewis et al., 2018). Wu et al. developed a sonic-based separation method that can directly isolate EVs from whole blood, greatly reducing the time required to extract EVs (Wu M. et al., 2017). These new technologies bring new opportunities for the diagnosis and treatment of diseases in the future.

### 2.3 Function of EVs

EVs were originally considered to be vesicles employed to expel excess transferrin receptor vesicles (Pan and Johnstone, 1983). With the development of the research, various functions of EVs were gradually revealed to the public. The vesicle structure of EVs can protect its internal transported substances from the interference of soluble substances such as proteases *in vivo*. At present, it is generally believed that EVs serve as carriers and play a big part in mediating information exchange between cells by transporting microRNAs (miRNAs), long non-coding RNAs (lncRNAs), tRNA-derived small RNAs (tsRNAs), proteins, lipids, and other substances (Mathivanan et al., 2012; Barile and Vassalli, 2017; Mathieu et al., 2019). These substances may be involved in the pathogenesis of different diseases. EVs of nasal epithelial cells in patients with chronic rhinosinusitis with nasal polyps contain differentially expressed proteins, which are mainly involved in epithelial remodeling through p53 and other pathways, leading to sinus mucosal remodeling (Zhou et al., 2020). EVs can carry *β*-Amyloid, prion, and *α*-synuclein, thus spread disease-causing proteins in the brain, which may be involved in Alzheimer’s disease progression (Nath et al., 2012; Arellano-Anaya et al., 2015; Lööv et al., 2016). Evidence shows that EV-associated miRNAs and lncRNAs play essential roles in the pathogenesis of osteoarthritis (OA), including OA diagnosis, pathogenesis, and treatment (Maehara et al., 2021; Miao et al., 2021). In addition, EVs are involved in many physiological processes, such as intracellular communication, signal transduction, transport of genetic materials, and modulation of immune response (Natasha et al., 2014). Evidence in previous studies indicates that EVs are also involved in the progression of diseases, including cancers, neurodegenerative diseases, and autoimmune diseases (Anderson et al., 2010), such as rheumatoid arthritis (RA), Sjogren’s syndrome (SS), and SLE. In this review, we summarize the latest progress and recent advances in EV research, therapeutic potential, and mechanism of EVs in the pathogenesis of SLE, as well as their clinical implications.

## 3 Role of EVs in Immune Function

It was not until 1996 that B cells were found to induce T cell responses by secreting EVs with major histocompatibility complex (MHC) class II, which indicated the relationship between EVs and immune regulation (Raposo et al., 1996). After that, EVs from other immunocytes, such as T cells, natural killer (NK) cells, and dendritic cells (DCs), have also been proven to mediate either immune stimulation or immune modulation (Gutierrez-Vazquez et al., 2013; Zhang et al., 2014; Jong et al., 2017; Reis et al., 2018).

### 3.1 EVs and Innate Immune Cells

Immune cell-derived EVs are involved in the regulation of the innate immune responses. EVs released by neutrophils, macrophages, NK cells, and DCs act on the innate immune system as pro-inflammatory mediators via paracrine messengers (Yanez-Mo et al., 2015).

#### 3.1.1 Neutrophils

Neutrophils are the most abundant leukocyte population in peripheral blood and are the first line of host defense against a wide range of infectious pathogens (Mayadas et al., 2014). In addition to regulating macrophage activation (Gasser and Schifferli, 2004), neutrophil-derived EVs have inhibitory effects on monocyte-derived DCs (Eken et al., 2008). These EVs modify the morphology of monocyte derived DCs (MoDCs) by inhibiting the formation of dendrites, downregulate their phagocytic activity and maturation, and inhibite the cytokine release of MoDCs, resulting in an attenuated capacity to stimulate T cell proliferation (Eken et al., 2008). Other studies identified several neutrophil-derived EV associated molecules which can influence DC and T cell function potentially, such as annexin A1 (Dalli et al., 2008; Gavins and Hickey, 2012) and arginase-1 (Leliefeld et al., 2015; Shen et al., 2017). What’s more, it was found that several proteases in neutrophil-derived EVs such as myeloperoxidase (MPO), elastase, cathepsin G and proteinase 3 may influence adaptive immunity (Hess et al., 1999; Gasser et al., 2003; Dalli et al., 2013; Timar et al., 2013; Slater et al., 2017).

#### 3.1.2 Macrophages

Another type of innate immune cells which is a rich source of EVs is macrophages (Wang et al., 2020). Macrophages are important phagocytic cells distributed in essentially all tissues, where they respond to a complex variety of regulatory signals to coordinate immune functions involved in tissue development, homeostasis, metabolism, and repair (Wynn and Vannella, 2016). EVs secreted by bacterially infected macrophages have a pro-inflammatory effect, which can induce the maturation of DCs and activate CD4^+^ and CD8^+^ T cells (Giri and Schorey, 2008; Ramachandra et al., 2010). Besides, these macrophage-derived EVs promote the release of multiple pro-inflammatory cytokines and chemokines (Singh et al., 2012). Furthermore, several studies characterized EVs content and their effects on uninfected macrophages which revealed that EVs released from infected macrophages holds a vital role in immune surveillance (Bhatnagar et al., 2007; Bhatnagar and Schorey, 2007).

#### 3.1.3 Natural Killer Cells

Natural killer (NK) cells are innate lymphoid cells with potent cytolytic function toward viral invasion and prevent survival or spread of tumor cells (Morvan and Lanier, 2016). NK cells have multiple activating receptors (e.g., NKG2D) and inhibitory receptors (e.g., killer-cell immunoglobulin-like receptors, KIRs), and the balance between these signals determines whether or not NK cells are activated (Fernandez-Messina et al., 2012; Sivori et al., 2019). NK cells are found to secrete EVs in a constitutive way and independent of their activation status (Lugini et al., 2012). Several studies reported that NK cell-derived EVs show cytotoxic activity against tumor cells (Fais, 2013; Zhu et al., 2017) and activate immune cells (Lugini et al., 2012).

#### 3.1.4 Dendritic Cells

As the sentinel antigen-presenting cells (APCs) of the immune system, dendritic cells (DCs) function as the link between innate and adaptive immunity, leading to either antigen-specific immunity initiation or tolerance (Steinman, 2012). Like DCs, EVs secreted by DCs were found to possess functional MHC-peptide complexes, costimulatory molecules, and other components that interact with immune cells (Thery et al., 1999; Thery et al., 2001; Thery et al., 2002a). EVs secreted by mature DCs contain class II MHC complexes and costimulatory molecules, which can directly interact with T cells to activate the immune system (Segura et al., 2005). On the other hand, EVs secreted by immature DCs can regulate the immune response, but do not function in direct T cell activation (Quah and O'Neill, 2005). In addition, studies have shown that DC-derived EVs can be absorbed by epithelial cells and promote the release of inflammatory mediators (MCP-1, IL-8, TNFα, RANTES) secreted by epithelial cells, suggesting that EVs promote immune-inflammatory response (Obregon et al., 2009).

### 3.2 EVs and Adaptive Immune Cells

The adaptive immune cells mainly include T and B lymphocytes.

#### 3.2.1 T Cells

DC-T cell interaction results in T cell activation. The interaction is transmitted from T cells to DCs via the transfer of EV-DNA, making DCs more resistant to infections (Torralba et al., 2018). EVs derived from activated CD3^+^ T cell together with IL-2 can modulate the activity of immune cells, including other T cells (Wahlgren et al., 2012). In addition, depending on their activation status, CD4^+^ T cells regulate the release of distinct vesicle subpopulations with various abilities to activate other untouched T cells (van der Vlist et al., 2012). T cell tolerance is shown due to EVs secreted by CD8^+^ suppressor T cells (Bryniarski et al., 2013). The protein expression profile of T cell EVs change substantially after different stimuli (activation vs apoptosis induction). Induction of apoptosis causes T cells to release more apoptotic bodies than exosomes, while activated T cells release exosomes and microvesicles both in lower amounts (Tucher et al., 2018). Studies have shown that Treg-derived EVs express immunomodulatory molecules (CD25, CD73, CTLA4), which have immunosuppressive effects and can regulate effector T cell proliferation and cytokine secretion to regulate immune response (Agarwal et al., 2014).

#### 3.2.2 B Cells

EVs derived from B cells exert a predominant role in antigen presentation and immunoregulation. B cell-derived EVs can induce antigen-specific MHC II-restricted T cell responses, suggesting antigen presentation capacities just like B cells (Raposo et al., 1996). Different types of antigens, carried by B cell-derived EVs, may dictate different types of immune responses (Hood, 2017). Recently, it has been suggested that B cell-derived EVs may have immunoregulatory functions which are independent of their ability to present antigen (Zhang et al., 2019). In addition, the role of different lymphocytes subsets (CD4^+^ T cells, CD8^+^ T cells, and NK cells) and DCs in CTL immune response to antigen presented on B-cell derived EVs has been described, demonstrating an complex interplay of cooperating lymphocytes for EV immunogenicity (Saunderson and McLellan, 2017).

## 4 Role of EVs in SLE and LN

### 4.1 EVs, SLE

EVs were found to be increased (Pereira et al., 2006; Sellam et al., 2009; Lee et al., 2016; Lopez et al., 2020) or decreased (Nielsen et al., 2011) in SLE patients compared to healthy controls. Proteins, mRNAs, miRNAs, lncRNAs, tsRNAs and other noncoding RNAs have been shown to be associated with EVs (Mathivanan et al., 2012; Barile and Vassalli, 2017; Mathieu et al., 2019). Recent studies have revealed that the ncRNAs play dominant roles in the pathogenesis of SLE (Long et al., 2018; Xie and Xu, 2018; Zhao et al., 2018; Chen et al., 2019; Liu et al., 2021). In this sense, miRNAs, lncRNAs, tsRNAs and proteins in SLE EVs might serve as biomarkers for disease diagnosis and therapeutic targets (Tan et al., 2016; Ortega et al., 2020).

#### 4.1.1 EVs, miRNA, SLE

MiRNA is a type of single-stranded non-coding RNA with a length of about 19–24 nucleotides, which can regulate the expression of many genes *in vivo* and participate in the pathogenesis of many diseases. Abnormal expression of circulating miRNAs in SLE patients have been found, and some of these miRNAs are related to clinical parameters (Carlsen et al., 2013; Ishibe et al., 2018). Circulating miRNAs are extracellularly secreted miRNAs circulating in the peripheral blood, which are either encapsulated by extracellular vesicles such as exosomes and microvesicles or bound to molecules such as the Argonaute protein or HDL cholesterol (Arroyo et al., 2011; Vickers et al., 2011). Tan et al. (2021) have reported that compared with healthy controls, serum EV miR-451a was decreased in SLE patients, which correlated with SLE disease activity and renal damage (Tan et al., 2021). Moreover, they found that EV shuttled miR-451a was involved in intercellular communication (Tan et al., 2021). Li et al. (2020) demonstrated that compared with healthy controls, serum EV miR-21 and miR-155 of SLE patients were up-regulated, whereas the expression of miR-146a was down-regulated (Li et al., 2020). Additionally, the expression of miR-21 and miR-146a were negatively correlated with anti-SSA/Ro antibodies and anti-dsDNA antibodies, respectively (Li et al., 2020). What’s more, both EV miR-21 and miR-155 expression presented positive correlations with proteinuria. These findings indicated that the expression levels of EV miR-21 and miR-155 might serve as potential biomarkers for the diagnosis of SLE. The aforementioned studies, however, have some limitations yet to be addressed. The mechanism underlying the reported dysregulation of the EV-associated miRNAs expression and the cell origin of the EVs remain unclear in the studies, which are performed based on relatively limited samples.

With the continual advances in this thread, the mechanism of EV-associated miRNAs in SLE pathogenesis has been revealed gradually. It is shown that miR-574, let-7b and miR-21 in plasma EVs can activate plasmacytoid DCs (pDCs) through the TLR7 pathway, enabling them to continuously produce IFN-α and proinflammatory cytokines, which may contribute to the pathogenesis of SLE (Salvi et al., 2018). Another study suggests that miR-146a could be internalized into mesenchymal stem cells (MSCs) via circulating EVs and participates in MSCs senescence in SLE patients by targeting TRAF6/NF-*κ*B signal pathway (Dong et al., 2019).

#### 4.1.2 EVs, lncRNA, SLE

LncRNA is another regulatory noncoding RNA longer than 200 nucleotides, capable of modulating many biological functions more specifically than miRNA. Aberrant circulating lncRNA expressions are found in SLE patients as well. Wu et al. (2017), Wu et al. (2019) found that plasma levels of GAS5, lnc7074 and lnc-DC were significantly reduced, whereas levels of linc0597, linc0640 and lnc5150 were elevated in SLE patients compared with those of healthy controls (Wu G.-C. et al., 2017; Wu et al., 2019). However, due to the complexity of its role, there is no literature regarding EV-associated lncRNA’s role in the pathogenesis of SLE. Thus, this promising research line is worthwhile to be investigated.

#### 4.1.3 EVs, tsRNA, SLE

Transfer RNAs (tRNAs) are a group of classic ncRNAs with a well-defined role in protein translation (Schulman and Abelson, 1988). tRNA-derived small RNAs (tsRNAs) are cleaved from precursor or mature tRNAs with a length of 18–40 nt and can be broadly classified into two main groups: tRNA halves and tRNA-derived fragments (tRFs) (Anderson and Ivanov, 2014). tRFs have been implicated to participate in diverse physiological processes and involved in many diseases through the protein synthesis by regulating mRNA expression (Zhu et al., 2018; Kim et al., 2020). In a recent study, Xu et al. (2020) found that tRNAs and tsRNAs were significantly differentially expressed in the PBMCs of SLE patients, compared with those of healthy donors, and the targeted genes of the differentially expressed tsRNAs were enriched in the signaling pathway involved in primary immunodeficiency, T cell receptor and Th cell differentiation, suggesting that tRNAs and tsRNAs play important roles in the pathogenesis of SLE (Xu et al., 2020). More precisely, Geng et al. (2021) showed that tRF-3009 was substantially over-expressed in CD4^+^ T cells of SLE patients than those of healthy donors. What’s more, tRF-3009 may be involved in SLE pathogenesis by modulation of IFN-α-induced CD4^+^ T cell oxidative phosphorylation (Geng et al., 2021). As for the EV, Dou et al. (2021) revealed that mesenchymal stem cell (MSC)-derived EV tsRNA-21109 inhibited the M1-type polarization of macrophages, offering a promising therapeutic strategy for SLE (Dou et al., 2021). Nowadays, Yang et al. (2021) found that tRF-His-GTG-1 was significantly upregulated both in serum of SLE without LN, and in serum EVs of SLE with LN compared with healthy controls, suggesting that it could be employed as a noninvasive biomarker for diagnosis and prediction of nephritis in SLE (Yang et al., 2021). Nonetheless, the exact mechanism underlying tsRNA-21109 and tRF-His-GTG-1 mediated SLE development remains yet to be elucidated.

#### 4.1.4 EVs, Protein, SLE

Proteins in EVs are also involved in the pathogenesis of SLE. Majer et al. (2019) found that UNC93B1 can be detected in RAW macrophage-derived EVs, it can limit TLR7 signaling and prevent TLR7-dependent autoimmunity in mice (Majer et al., 2019). Moreover, UNC93B1 mutation can enhance the TLR7 signaling pathway, leading to the development of autoimmune diseases (Majer et al., 2019). More recently, Chuang et al. (2021) proved that bactericidal/permeability-increasing protein (BPI) is a negative regulator of Treg differentiation (Chuang et al., 2021). They identified the overexpression of BPI in T cells and T cell-derived EVs contributed to autoimmune responses through both intrinsic (inhibition of Treg population) and extrinsic (induction of inflammatory EVs) pathways, which might be a biomarker and a pathogenic factor for SLE (Chuang et al., 2021). And just this month, they reported another EV-associated protein, Eosinophil Cationic Protein (ECP, also named RNase 3), which was overexpressed in SLE T cell-derived EVs (Chuang et al., 2022). What’s more, ECP overexpression in T cells resulted in an increase of inflammatory responses and T-cell activation. Notably, ECP-containing EVs from T cells led to tissue inflammation of the recipient mice. These results suggest that ECP-overexpressing T cells or ECP-containing EVs may play an important role in SLE pathogenesis (Chuang et al., 2022). Nevertheless, except for the small sample size, it would be challenging yet essential to dig into the fundamental mechanisms of BPI/ECP-induced inflammation via EVs in the future.

### 4.2 EVs, LN

Lupus nephritis (LN) is one of the most devastating manifestations of SLE, and a primary cause of morbidity and mortality of SLE (Hahn et al., 2012). At present, renal biopsy is still the gold standard for diagnosis and evaluation of residual nephron function. Renal puncture, however, presents many perilous complications and can only reflect the state of a small part of the renal tissue. Therefore, pursuing a non-invasive and sensitive diagnostic method is urgently needed (Ortega et al., 2010; Morell et al., 2021). Over the past few years, changes in urinary miRNAs have been reported in LN patients, and its expression may be relevant to disease activity (So et al., 2021).

#### 4.2.1 EVs, miRNA, LN

Urinary EV-associated miRNAs are promising novel markers for the diagnosis and prognosis of disease, and also clinical outcomes. Lv et al. (2013) showed that high levels of miRNA were confined to urinary EVs in patients with a diversity of chronic diseases (Lv et al., 2013). Urinary EV in lupus nephritis was first described in 2014, Ichii et al. (2014) first found that in patients with lupus nephritis, the expression level of miR-26a in urinary EVs was significantly higher than that in the control group, and miR-26a expression was related to podocyte injury, suggesting that miR-26a can be used as a direct biomarker for podocyte injury in autoimmune glomerulonephritis (Ichii et al., 2014). Solé et al. (2015) showed reduced expression level of miR-29c in LN patients compared with healthy controls, and its level in urinary EVs was negatively correlated with the histological chronicity index and glomerular sclerosis, indicating that miR-29c level could be used as a novel non-invasive marker for predicting histological fibrosis of LN (Solé et al., 2015). As the research proceeds, the mechanism of urinary EV-associated miRNAs in LN pathogenesis has been gradually revealed. Recently, their team revealed that miR-21 and miR-150 were substantially up-regulated while miR-29c was down-regulated in the urinary EVs of LN patients, and their expression was strongly correlated with renal chronicity. They also demonstrated that these miRNAs promoted renal fibrosis through SP1 and Smad3/TGFβ signaling pathway (Solé et al., 2019). And a more recent study by their team found that the overexpression of urinary EV miR-135b-5p, miR-107, and miR-31 could meliorate renal disease by inhibiting HIF-1α, suggesting their potential to become early markers for predicting clinical response in LN (Garcia-Vives et al., 2020). Perez-Hernandez et al. (2015) confirmed that urinary miRNAs were contained mainly in EVs, and they reported an impressive increase in miRNA-146a in urinary EVs in patients with active lupus nephropathy, implying that miRNA-146a in EVs may be able to distinguish SLE patients with active LN from control group or SLE patients in absence of LN (Perez-Hernandez et al., 2015). Recently, their group identified a protective role that urinary EV miR-146a played in LN progression through negative regulation of inflammation by suppressing the TRAF6 axis (Perez-Hernandez et al., 2021). Furthermore, it has been evidenced that Type IV lupus nephritis with cellular crescent (LNIV-CC) has a unique urinary EV-associated miRNA expression profile, and urinary EV miR-3135b, miR-654-5p and miR-146a-5p are candidate biomarkers for LNIV-CC (Li et al., 2018). In addition, another study discovered that compared with inactive disease, let-7a and miR-21 in urine EVs were significantly down-regulated in LN patients with active disease. Interestingly, their expression increased after the entire course of treatment, indicating that urinary EV-related miRNA, let-7a and miR-21, may be leveraged to guide the clinical stage of LN patients (Tangtanatakul et al., 2019). The above findings indicate that miRNAs in urinary EVs may have great potential to serve as biomarkers in LN diagnosis and monitoring.

#### 4.2.2 EVs, Protein, LN

Proteins in urine EVs are also involved in the pathogenesis of LN. Gudehithlu et al. (2019) found that urine EV ceruloplasmin (CP) was increased in LN patients. What’s more, in biopsied cases, CP was strongly localized to kidney tubules, suggesting that the CP found in urine EVs came from the kidney. Moreover, in mouse models, urine EV CP were observed to increase prior to proteinuria, indicating it could be an early biomarker to diagnose kidney disease (Gudehithlu et al., 2019).

These findings strongly suggest the potential role of urinary EVs as a non-invasive biomarker in the diagnosis and treatment of LN. Multiple limitations of the present studies, however, need to be acknowledged and are summarized as follows. Firstly, studies involving a reasonably larger patient cohort are necessary for further analyzing and validating these findings, especially for diagnosis. Secondly, most of the research works described above are cross-sectional studies. The longitudinal studies such as during disease flare or before and after treatment are also needed to further extend these findings. Thirdly, the thorough comparative analysis between the reported urinary EV-associated miRNAs/protein and the existing inflammatory and clinical markers of disease is missing and thus worthwhile to be performed in the further. Fourthly, the differentially expressed urinary EV-associated miRNAs/protein is delivered from various kidney cell types. Further study to characterize specific cell types that contribute to the dysregulation of miRNAs/protein in urine EVs is thus demanded. What’s more, the present studies lack functional experiments at molecular or cellular level to verify the association between miRNAs/protein and LN. All these imperfections are likely due to the low recovery ration, low yield and purity of EV extraction as well as the immature technology of EV transfection and infection.

## 5 Conclusion

In recent years, EVs have emerged as an important endogenous “nanovehicles” for carrying and transferring molecular mediators such as nucleic acids, proteins, and bioactive lipids for intercellular communications and signal transduction. Besides, it is well established that EVs are involved in a multitude of physiological and pathological processes, such as immune response, antigen presentation, cell differentiation, cell migration, and tumor invasion. A rapidly expanding body of evidence indicates that the presence of EV-specific patterns and their cargo play crucial physiological and pathological roles in SLE. In this context, miRNAs, tsRNAs, and proteins transported into serum/plasma/urinary EVs are correlated with glomerular damage, SLE disease activity, clinical stage and response, proteinuria as well as the severity of renal fibrosis in lupus nephritis. In this article, we provide the up-to-date survey of relevant literature evidencing that EVs have great potential in SLE disease diagnosis, prediction, prognosis, and targeted treatment (Figure 1 and Table 1). However, the underlying pathophysiological mechanisms of EVs in SLE pathogenesis and their functionality as therapeutic agents or targets are not fully understood. Future investigations into the exact mechanisms of EVs in SLE will undoubtedly bring new breakthroughs for SLE disease diagnosis and therapies.
